# Modeling the Hydrodynamics of Phloem Sieve Plates

**DOI:** 10.3389/fpls.2012.00151

**Published:** 2012-07-13

**Authors:** Kaare Hartvig Jensen, Daniel Leroy Mullendore, Noel Michele Holbrook, Tomas Bohr, Michael Knoblauch, Henrik Bruus

**Affiliations:** ^1^Department of Micro- and Nanotechnology, Technical University of DenmarkKongens Lyngby, Denmark; ^2^Department of Physics, Technical University of DenmarkKongens Lyngby, Denmark; ^3^Department of Organismic and Evolutionary Biology, Harvard UniversityCambridge, MA, USA; ^4^School of Biological Sciences, Washington State UniversityPullman, WA, USA

**Keywords:** biological fluid dynamics, hydraulic resistance, phloem, sieve plate, sugar transport in plants

## Abstract

Sieve plates have an enormous impact on the efficiency of the phloem vascular system of plants, responsible for the distribution of photosynthetic products. These thin plates, which separate neighboring phloem cells, are perforated by a large number of tiny sieve pores and are believed to play a crucial role in protecting the phloem sap from intruding animals by blocking flow when the phloem cell is damaged. The resistance to the flow of viscous sap in the phloem vascular system is strongly affected by the presence of the sieve plates, but the hydrodynamics of the flow through them remains poorly understood. We propose a theoretical model for quantifying the effect of sieve plates on the phloem in the plant, thus unifying and improving previous work in the field. Numerical simulations of the flow in real and idealized phloem channels verify our model, and anatomical data from 19 plant species are investigated. We find that the sieve plate resistance is correlated to the cell lumen resistance, and that the sieve plate and the lumen contribute almost equally to the total hydraulic resistance of the phloem translocation pathway.

## 1 Introduction

**Figure 1:**
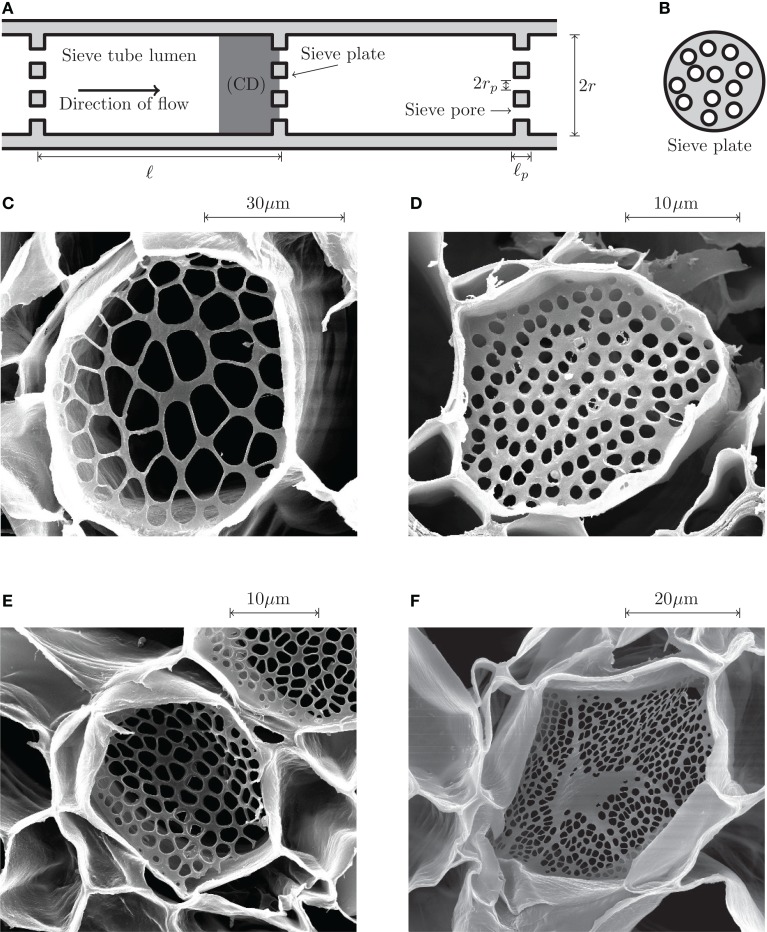
**Phloem sieve tube geometry**. **(A)** Schematics of a sieve tube. Adjacent sieve tube cells of length ℓ and radius *r* are separated by thin sieve plates of thickness ℓ_*p*_ perforated by small holes of radius *r_p_* known as sieve pores. The computational domain (CD) used in the numerical simulations is highlighted in dark gray. **(B)** Schematic end view of a sieve plate. On average, about 50% of the sieve plate area is covered by open pores. **(C–F)** Scanning electron microscope (SEM) images of sieve plates. **(C)** *Cucurbita maxima* (Squash). **(D)** *Phyllostachys nuda* (Bamboo). **(E)** *Phaseolus vulgaris* (Green bean). **(F)** *Ricinus communis* (Castor bean). As shown in Figure 2, the sieve pore radius *r_p_* is normally distributed with a standard deviation σ*_p_* of approximately 25% of the mean value ${\bar{r}}_{p}$ See Mullendore et al. (2010) for details on SEM imaging.

The phloem vascular system of higher plants can be thought of as a combination of the circulatory and nervous systems found in animals, distributing photosynthetic products, and carrying signals throughout the plant (Taiz and Zeiger, 2002). Transport occurs through narrow elongated cylindrical cells, known as sieve tube elements, lying end-to-end forming a microfluidic network spanning the entire length of the plant. Adjacent sieve tube elements are separated by a sieve plate, a thin plate perforated by a large number of tiny pores, as shown in Figures 1A,B.

The sieve tube structure has a pronounced effect on the flow due to the viscous drag imposed by the presence of the cell walls, the cell organs, the sieve plates, and other parietal materials (Crafts and Crisp, 1971; Thompson and Holbrook, 2003). Until recently, limited anatomical resolution has made detailed studies of the anatomy difficult. Using a novel method to clear cells of cytoplasmic constituents, Mullendore et al. (2010) investigated the detailed structure of cell walls and sieve plates using scanning electron microscopy. Their study provides vital insight into the geometry of pores, plates, and sieve elements, as shown in Figures 1C–F. However, the effect of the pores, plates, and sieve element walls on the flow of liquid inside the cells remains uncertain at best. More precise quantitative characterizations of the flow and resulting hydraulic resistances are needed for evaluating phloem function.

Our aim with this study is to create a theoretical model to quantify the effect of the sieve plates on the phloem flow and to develop a computational fluid mechanics procedure for studying the qualitative and quantitative properties of the flow near a sieve plate.

## 2 Quantitative Analysis of Flow in Sieve Tubes

### 2.1 Characteristic flow properties

**Figure 2:**
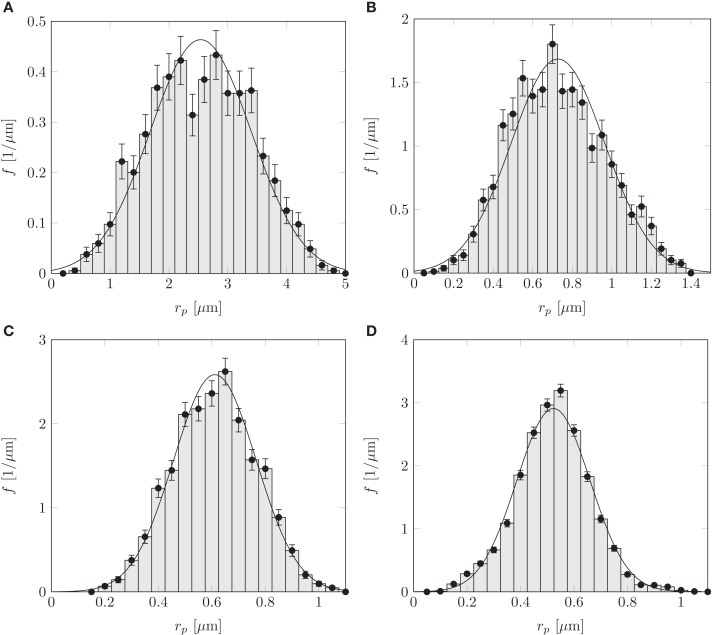
**Normalized histograms showing sieve pore radius *r_p_* distributions for (A) *Cucurbita maxima*, (B) *Phaseolus vulgaris*, (C) *Solanum lycopersicum*, and (D) *Ricinus communis***. The solid lines are normal distributions fitted to the histograms, giving mean ${\bar{r}}_{p}$ and standard deviation σ*_p_* as shown in Table A1 in Appendix. Data from Mullendore et al. (2010). For each species, 5–20 sieve plates were sampled with a total of 529–6281 sieve pores per species.

The flow of sugars in the phloem is believed to be driven by differences in hydrostatic pressure between source (leaves) and sink tissues (e.g., roots or fruits). These pressure differences are generated by differences in the chemical potential between source and sink tissues, and drive flows with velocities of the order *u* ≃ 1 m/h = 280 μm/s (Mullendore et al., 2010). The characteristics of the phloem cells and sieve plates vary among species as summarized in Table A1 in Appendix A, but the radius *r* of the sieve tubes is typically 10 μm, while the pore radius *r_p_* ≃ 1 μm is often an order of magnitude smaller. Within a single sieve plate, the pore radii vary, see, e.g., Figure 1C. Examining data from Mullendore et al. (2010), we find as shown in Figure 2 that the pore radii are normally distributed with mean ${\bar{r}}_{p}$ and standard deviation σ*_p_* given by the relation

(1) $$\sigma_{p}≃0.25\;{\bar{r}}_{p}.$$

The sugar concentration *c* of the aqueous solution flowing in the tube is typically in the range 0.1−1 M, and consequently we may take the viscosity η of the aqueous sugar solution to be a few times that of water, say, η≃ 2 mPas, while the density *ρ* ≃ 10^3^ kg/m^3^ is almost unaffected. Basic features of the flow may be revealed by considering the magnitude of the Reynolds number *Re* which characterizes the relative importance of inertial and viscous forces. In the sieve tube lumen – far away from the pores – it is given by

(2) $$Re_{L}=\frac{\rho ur}{\eta }≃1.4\times 10^{-3}.$$

Near the sieve plate, the flow speed increases as the liquid passes through the sieve pores. If we take the characteristic pore flow velocity to be *u_p_*, volume flux conservation dictates that $u\pi r^{2}=N\pi r_{p}^{2}u_{p},$ where *N* is the number of pores. Introducing the pore covering fraction $\varphi =Nr_{p}^{2}\text{/}r^{2},$ which is typically 0.5, or 50%, we find that $u_{p}=u/\varphi ≃2u$. The Reynolds number *Re_p_* associated with the flow in the pores is thus 5 times smaller than in the cell lumen

(3) $$Re_{P}=\frac{\rho u_{p}r_{p}}{\eta }≃2.8\times 10^{-4}.$$

At these low Reynolds numbers, viscous forces dominate the flow, and the relevant equations of motion for the flow velocity **u** and pressure *p* are the Stokes equation and the continuity equation,

(4) $$\nabla p=\eta \nabla^{2}\text{u},\;\;\nabla \cdot \text{u}=0.$$

The Stokes equation is valid for describing flows in channels wider than about 10 nm (Koplik and Banavar, 1995) well within the range of pores sizes considered in the present paper. Several workers have studied flow through small pores at low Reynolds numbers experimentally. Johansen (1930) found that for *Re_p_* ≤ 30 the flow is laminar and left-right symmetric close to the pore. In fact, it remains laminar until *Re_p_* ≃ 10^3^, but symmetry is broken above *Re_p_* = 30. Johansen also found that the length ℓ* of the region upstream affected by the presence of the pore is roughly equal to the pore diameter, which is in agreement with standard theory for pipe flows predicting an entrance-length roughly equal to the pipe radius and independent of flow rate at these low Reynolds numbers (Lautrup, 2011; Bruus, 2012).

Due to the complex nature of the sieve plates shown in Figures 1C–F, obtaining analytical solutions of the Stokes equation (4) for the flow close to the sieve plates is not generally possible. Idealized versions of the flow, however, have been studied extensively in situations where the pore length ℓ_*p*_ is very small compared to the pore radius *r_p_* (Sampson, 1891; Roscoe, 1941; Hasimoto, 1958; Wang, 2004; Jeong and Choi, 2005) and for pores of length comparable to the pore radius *r_p_* (Weissberg, 1962; Dagan et al., 1982). A result first shown by Weissberg (1962), and later by Dagan et al. (1982), is that the hydraulic resistance R* of a single pore of radius *r_p_* in an infinite plane of thickness ℓ_*p*_ can be approximated by the addition formula for serial hydraulic resistors,

(5) $$R^{*}=\frac{8\eta \ell_{p}}{\pi r_{p}^{4}}+\frac{3\eta }{r_{p}^{3}}.$$

Here, the first term on the right-hand side is the well-know formula for the resistance of a cylindrical pipe of length ℓ_*p*_ and radius *r_p_*, while the second term represents the resistance of a pore in an infinite plate of zero thickness as first derived for a circular pore by Sampson (1891) and later generalized to other shapes and arrays of pores by Roscoe (1941) and Hasimoto (1958). As expected, equation (5) performs worst when the pore diameter is comparable to its length ℓ_*p*_ ≃ 2*r_p_*, but even then the error is only around 1%. The hydrodynamic interaction with neighboring pores was investigated semi-analytically by Wang (2004), who showed that the resistance in the limit ℓ_*p*_ ≪ *r_p_* differed by less than 10% from that found in equation (5) for the covering fractions φ ≤ 50% found in plants (see Wang, 2004; Figure 2). Finally, one must take into account that the sieve plate is embedded in a larger circular tube. The effect of the surrounding channel walls on the resistance of the pore was studied by Jeong and Choi (2005) and shown to lead an error of less than 2% for *r_p_*/*r* ≤ 0.2.

### 2.2 Hydraulic resistor theory

Having established the basic characteristics of the flow in Sec. 2.1, it appears likely that the formalism of hydraulic resistor theory will be able to capture essential features of the flow in phloem sieve tubes. Indeed, the use of hydraulic resistance models based on Kirchhoff’s circuit laws (Bruus, 2008) in studies of phloem physiology dates back at least as far as Crafts and Crisp (1971). Mathematically, both phloem translocation and xylem transpiration would also seem to be homologous with stomatal transpiration, in which resistive modeling has long been used (Parkhurst, 1994).

In the following, we derive a general expression for the resistance of a sieve tube/sieve plate system, and compare it to numerical simulations of real and idealized sieve tubes. When calculating the hydraulic resistance *R* of a single phloem sieve tube shown in Figure 1A, we may think of it as two resistors coupled in series,

(6) $$R=R_{L}+R_{P},$$

where *R_L_* is the resistance of the cell lumen and *R_P_* is the resistance of the sieve plate. The flow velocity *u* can be determined from the hydraulic resistance *R* as *u* = Δ*p*/(*AR*), where Δ*p* is the pressure differential driving the flow and *A* = π*r*^2^ is the cross section area of the tube. We approximate the cell lumen by a cylindrical tube of length ℓ and radius *r*, whereby *R_L_* becomes

(7) $$R_{L}=\frac{8\eta \ell }{\pi r^{4}}.$$

The numerical pre-factor of the lumen resistance depends on the cross section shape of the channel (a circle leads to the value 8/π), whereas ηℓ*r*^4^ is a cross section-independent factor common to all straight channel (see e.g., Bruus, 2008).

Several different methods for calculating the sieve plate resistance *R_P_* have been proposed (see e.g., Thompson and Holbrook, 2003; Mullendore et al., 2010). Generally, the idea has been to consider the plate as a collection of individual pores coupled in parallel, whereby the hydraulic resistance *R_P_* of the pores can be expressed as

(8) $$R_{p}={\left(\sum_{i=1}^{N}R_{p,i}^{-1}\right)}^{-1},$$

where *R_p,*i*_* is the hydraulic resistance of the *i*th pore, which is general are unequal as the pore radii *r_p,*i*_* differ. The paralleling of pore resistances has also been used to characterize flow in the xylem vascular system in which structures similar to sieve plates are found (Pickard, 1982).

If the first term in equation (5) dominates the resistance, i.e., if (3π/8)*r_p_*/ℓ_*p*_ ≃ *r_p_*/ℓ_*p*_ ≪ 1, we may follow Mullendore et al. (2010) and write for the resistance $R_{p,i}=8\eta \ell_{p}∕\left(\pi r_{p,i}^{4}\right)$. Another approach, used by Thompson and Holbrook (2003), is to ignore variations in the pore radii *r_p,*i*_* and simply use the arithmetic mean radius ${\bar{r}}_{p}=\frac{1}{N}\sum_{i=1}^{N}\;r_{p}$ for all pores in equation (5) while keeping terms of order $1∕r_{p}^{3}$ in equation (5). This yields $R_{p,i}=\frac{8\eta \ell_{p}}{\pi {\bar{r}}_{p}^{4}}+\frac{3\eta }{{\bar{r}}_{p}^{3}},$, which is independent of the pore index *i*, and equation (8) becomes $R_{p}=\frac{1}{N}\left(\frac{8\eta \ell_{p}}{\pi {\bar{r}}_{p}^{4}}+\frac{3\eta }{{\bar{r}}_{p}^{3}}\right).$

It is not clear how the aforementioned approximations affect the accuracy of the calculated sieve plate resistance *R_P_*. As discussed in Sec. 3.2, they do in fact introduce significant errors in the estimates of the resistance. This is most like due to (i) that the sieve plate thickness ℓ_*p*_ is often comparable to the pore radius *r_p_* (see Table A1 in Appendix A), and (ii) that considerable variation in pore sizes are often found within a single plate, see Figure 1C and Sec. 2.

Making no such approximations, we propose to write the pore resistance *R_p,i_* as

(9) $$R_{p,i}=\frac{8\eta \ell_{p}}{\pi r_{p,i}^{4}}+\frac{3\eta }{r_{p,i}^{3}},$$

such that the total resistance becomes

(10) $$R=\frac{8\eta \ell }{\pi r^{4}}+{\left(\sum_{i=1}^{N}{\left(\frac{8\eta \ell_{p}}{\pi r_{p,i}^{4}}+\frac{3\eta }{r_{p,i}^{3}}\right)}^{-1}\right)}^{-1}.$$

For much of the data in the literature we do not have access to the full set of measured pore radii, and consequently we cannot evaluate the sum over the individual pores in equation (10) directly. From the five species studied in Mullendore et al. (2010 see Table A1 in Appendix A; Figure 2) we know, however, that the pore radii *r_p,i_* are normally distributed with mean ${\bar{r}}_{p}$ and standard deviation $\sigma_{p}≃0.25\;{\bar{r}}_{p}.$. This allows us to write the sum in equation (10) in terms of the normal probability density function $p(r_{p})=\exp[{\left({\bar{r}}_{p}-r_{p}\right)}^{2}/(2\sigma_{p}^{2})]/\sqrt{2\pi \sigma_{p}^{2}},$ as

(11) $$\begin{array}{c} R≃\frac{8\eta \ell }{\pi r^{4}}+\frac{\eta }{N}\left[\int \frac{1}{\sqrt{2\pi \sigma_{p}^{2}}}\exp\left(-\frac{{\left({\bar{r}}_{p}-r_{p}\right)}^{2}}{2\sigma_{p}^{2}}\right)\right.\\ \;\;\;{\left.\times {\left(\frac{8\ell_{p}}{\pi r_{p}^{4}}+\frac{3}{r_{p}^{3}}\right)}^{-1}\;\text{d}r_{p}\right]}^{-1}\\ =\frac{8\eta \ell }{\pi r^{4}}+\frac{\eta }{N}\frac{1}{{\bar{r}}_{p}^{3}}\left[\int_{0}^{\infty }\frac{1}{\sqrt{2\pi \beta^{2}}}\exp\left(-\frac{{\left(1-\xi \right)}^{2}}{2\beta^{2}}\right)\right.\\ \;\;\;\;{\left.\times {\left(\frac{8\alpha }{\pi \xi^{4}}+\frac{3}{\xi^{3}}\right)}^{-1}\;\text{d}\xi \right]}^{-1}, \end{array}$$

where we have introduced the re-scaled pore radius $\xi =r_{p}/{\bar{r}}_{p}$ and the parameters $\alpha =\ell_{p}/{\bar{r}}_{p}$ and $\beta =\sigma_{p}/{\bar{r}}_{p}$. Several additional parameters in the problem, such as the plate thickness ℓ_*p*_, are most likely also normally distributed, and a more accurate version of equation (11) could include this. In the case of equation (11), however, variations in the pore radius is clearly most important since it enters to the power 3 and 4, and thus will contribute more significantly to the variation of the total hydraulic resistance *R* than the other parameters.

### 2.3 Numerical flow simulations

**Figure 3:**
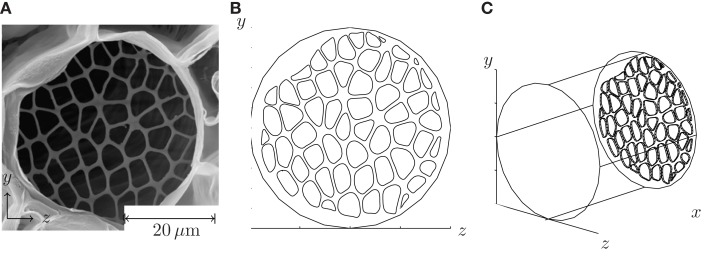
**Procedure for setting up numerical simulations of flow in sieve tubes**. In **(A)**, a SEM image of a sieve plate found in *Cucurbita maxima* is shown. From Mullendore et al. (2010), reproduced with permission. **(B)** Front view of the pore structure extracted from **(A)**. **(C)** The plate has been place inside a cylindrical tube, cf. the computational domain shown in Figure 1A.

We have solved the equations of motion equation (4) numerically to be able to test our theoretical prediction for the hydraulic resistance given in equations (10) and (11), and to qualitatively study the flow pattern inside phloem sieve tubes. Below and in Figure 3, we have briefly outlined our procedure, and more details can be found in Appendix A.

First, a geometry was chosen based on a SEM image of an actual biological sieve plate. The plate structure was extracted and encoded numerically as the end-wall in the cylindrical computational domain. The velocity and pressure fields was subsequently determined from equation (4) using the no-slip boundary condition at all the side-walls.

To evaluate the accuracy of equations (10) and (11), flow through an idealized sieve plate with circular pores of random (non-overlapping) positions and normally distributed sizes was also studied. As in Figure 3 this plate structure was placed at the end-wall of the same computational domain. The flow and pressure fields were determined numerically as a function of the geometric parameters of the problem: the sieve tube length ℓ, the sieve plate thickness ℓ_*p*_, the mean sieve pore radius ${\bar{r}}_{p}$, the sieve pore radius standard deviation σ*_p_*, and the covering fraction Φ. The numerical simulations were conducted using non-dimensional variables and the following non-dimensional parameters were used (see also Table 1).

**Table 1:** **Parameters ranges used in the numerical study**.

Parameter	Expression	Value
Pore-plate aspect ratio	$\alpha =\frac{\ell_{p}}{{\bar{r}}_{p}}$	0.4–1.2
Relative pore standard deviation	$\beta =\frac{\sigma_{p}}{{\bar{r}}_{p}}$	0.2–0.4
Cell aspect ratio	$\gamma =\frac{l}{r}$	10–100
Pore-cell aspect ratio	$\delta =\frac{{\bar{r}}_{p}}{r}$	0.05–0.2
Covering fraction	$\varphi =\frac{\sum_{i=1}^{N_{p}^{v}}r_{p,i}^{2}}{r^{2}}$	0.1–0.6

(12) $$\alpha =\frac{\ell_{p}}{{\bar{r}}_{p}},\;\beta =\frac{\sigma_{p}}{{\bar{r}}_{p}},\;\gamma =\frac{\ell }{r},\;\delta =\frac{{\bar{r}}_{p}}{r},\;\phi =\frac{\sum_{i}^{N}\;r_{p,i}^{2}}{r^{2}}.$$

The simulation allowed us to determine the hydraulic resistance *R*_num_ numerically as a function of these parameters. From the convergence analysis described in Appendix A, we estimate the relative error in *R*_num_ to be less than 5%.

## 3 Results

### 3.1 Qualitative properties of flow in sieve tubes

**Figure 4:**
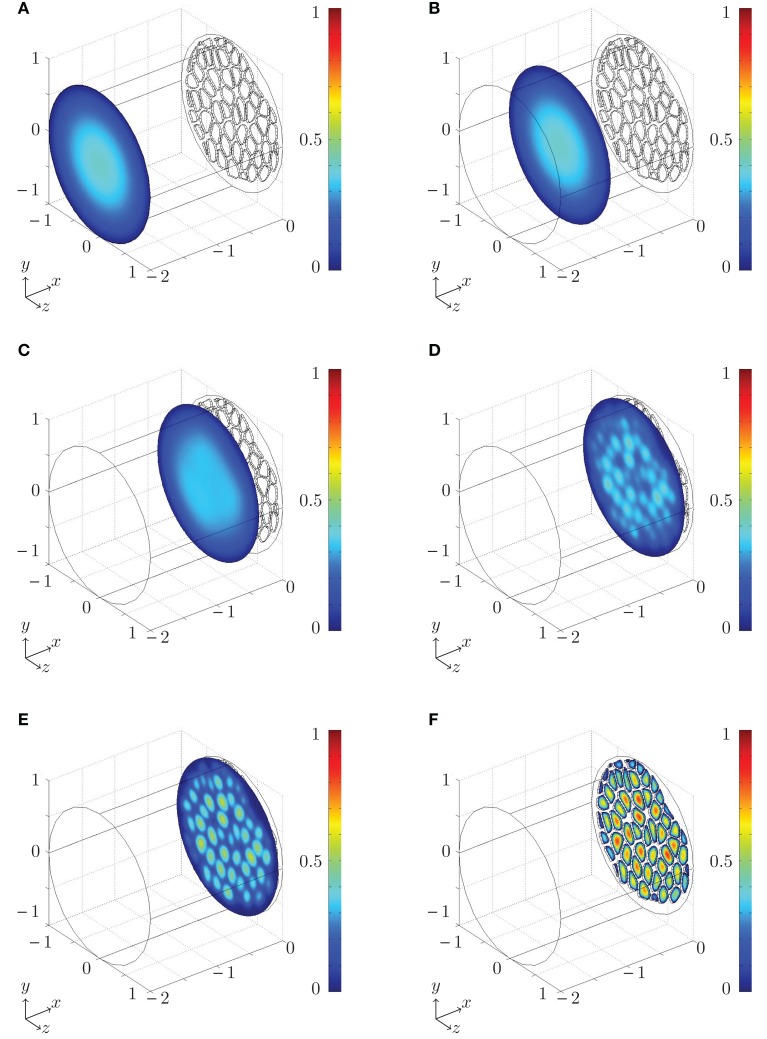
**Numerical simulation of the flow close to a *Cucurbita maxima* sieve plate set up using the procedure described in Sec. 2.3 and Figure 3. Color plot of the magnitude of the flow velocity |u| (from blue = 0 to red = 1 in arbitrary units)**. The liquid is moving from left to right. In **(A–F)**, the non-dimensional distance *x* from the sieve plate (in units of the tube diameter) at which the cross sections are taken is *x* = −2, −1.25, −0.36, −0.13, −0.07, 0.025. The flow profile in **(A,B)** is the familiar parabolic profile found in conventional pipe flows. **(C–E)** The flow is distorted progressingly more for diminishing distance to the sieve plate where **(F)** the fluid passes through the pores. See Movie S1 in Supplementary Materials and Appendix B for further details.

A numerical example of the flow close to a sieve plate is shown in Figure 4 and Movie S1 in Supplementary Materials, where color plots of the magnitude |***u***| of the flow velocity ***u*** is shown. Far away from the plate we find the familiar parabolic profile, characteristic of a conventional pressure-driven pipe flow in a straight cylindrical tube. Closer to the sieve plate the flow is disturbed by the presence of the plate, because the fluid must change direction in order to pass through the pores. The distance from the plate at which the flow is significantly disturbed is seen to be a few pore diameters, in good agreement with the experiments carried out by Johansen (1930).

### 3.2 Testing the theoretical predictions of the hydraulic resistor model

**Figure 5:**
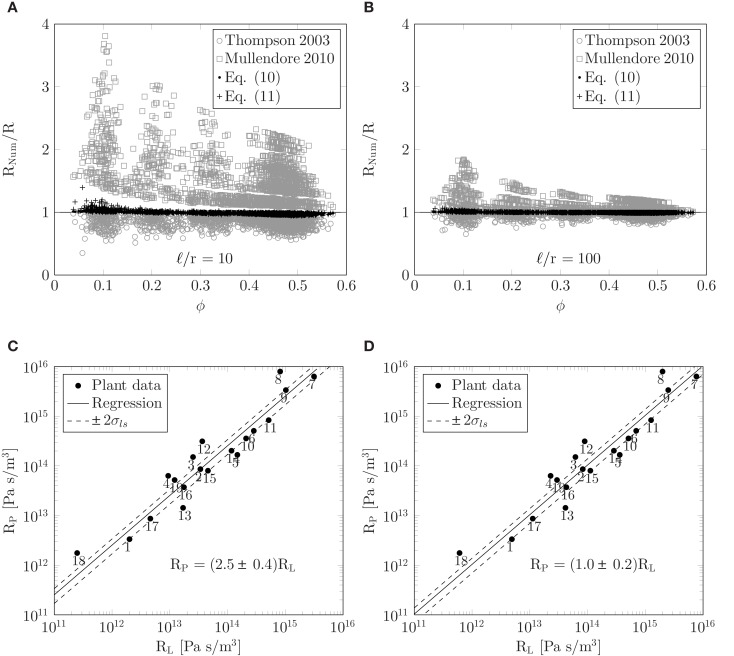
**(A,B)** Testing the theoretical predictions of the resistor model. Resistance ratio *R*_num_/*R* plotted as a function of the covering fraction Φ for ℓ/*r* = 10 **(A)** and ℓ/*r* = 100 **(B)**. The numerical resistance *R*_num_ was determined from numerical simulations of flow in idealized sieve plates with circular pores for 1980 combinations of the parameters given in equation (12) covering the ranges given in Table 1. The predicted resistance *R* was calculated from equations (10) and (11) and with the methods from Thompson and Holbrook (2003) and Mullendore et al. (2010), as described in Sec. 2.2. **(C–D)** Relationship between lumen and plate resistance. Sieve plate resistance *R_P_* plotted as a function of the lumen resistance *R_L_* calculated from equation (11) using data from Table A1. In **(C)** the entire sieve tube lumen is assumed to be open to flow. Solid line is least squares fit to *R_P_* = *kR_L_* which yields *k* = 2.5 ± 0.4 (*r*_corr_ = 0.78, *N* = 19). In **(D)** 20% of the sieve tube lumen radius is assumed to be blocked by organelles, i.e., the effective radius open to flow is 0.8*r*. Solid line is least squares fit to *R_P_* = *kR_L_* which yields *k* = 1.0 ± 0.2 (*r*_corr_ = 0.78, *N* = 19). To indicate the width of the least square fits, dashed lines in **(C,D)** show *R_P_* = (*k* ± 2σ*_ls_*)*R_L_*, where σ*_ls_* is the calculated uncertainty in *k*.

We conducted numerical simulations of flow through sieve plates with 1980 different combinations of the parameters given in equation (12) covering the ranges given in Table 1. In each case, the resistance *R*_num_ was determined numerically. Figures 5A,B show the ratio of *R*_num_ and the resistances *R* predicted by equations (10) and (11) as a function of covering fraction Φ. We find deviations of less than 10% between theoretical and numerical results over the whole parameter space. We attribute the remaining errors to the hydrodynamic interaction between the pores which is not included in the present model. We also show *R*_num_/*R* calculated with methods from Thompson and Holbrook (2003) and Mullendore et al. (2010), and note that for the large cell aspect ratio ℓ/*r* = 100 in Figure 5B these methods perform fairly well, while for the shorter cell aspect ratio ℓ/*r* = 10 in Figure 5A they are less accurate. These deviations are presumably due to variations in sieve pore sizes and to the contribution of the finite thickness of the sieve plate to the resistance. Finally, Figures 5A,B shows that the positioning of the pores in the plate does not play a significant role when determining the hydraulic resistance.

### 3.3 The relationship between lumen and plate resistance

Having established equation (11) as an approximate expression for the resistance of the sieve tube, we can now apply it to the data in Table A1. To best interpret the results, we calculate the lumen and plate resistance separately and compare their magnitudes. In Figure 5C, a log-log plot is shown of the sieve plate resistance *R_P_* versus the lumen resistance *R_L_*. Both were calculated from equation (11) using data from Table A1 under the assumption that η = 2 × 10^−3^ Pa s. For data points 6–19 we assume that σ*_p_* *=* 0.25*r_p_*. We observe that *R_P_* is linearly proportional to *R_L_* over four orders of magnitude, and a least-squares regression yields

(13) $$R_{P}=\left(2.5\pm 0.4\right)R_{L},$$

with a correlation coefficient of *r*_corr_ = 0.78. This implies that the presence of sieve plates increases the hydraulic resistance of the entire sieve tube element by a factor of ∼3.5, or put differently, the presence of the sieve plates is equivalent to an increase of the effective viscosity by a factor ∼3.5 in a tube without the sieve plate. The correlation in equation (13) assumes that the sieve elements are completely open to flow. If instead we assume that 20% of the sieve tube radius is blocked, i.e., that the effective radius of the tube is 0.8*r*, we find a one-to-one relationship between the two resistances *R_P_* = (1.0 ± 0.2)*R_L_* as shown in Figure 5D, such that the presence of the sieve plate is equivalent to an increases in the effective viscosity in a plate-less tube by a factor ∼2.

While the trend of the plots in Figures 5C–D is clear, it is obvious that many effects are influencing the relation between plate and lumen resistance. As an example it is interesting to consider, say, plant no. 13 which is *Sabal palmetto*, a palm tree that lies some distance from the *R_P_* ∼ *R_L_* line. In this plant the sieve tubes are found inside the stem, rather than right under the bark which is usually the case in trees, and are thus mechanically protected against insects and other predators (Parthasarathy and Tomlinson, 1967; Thompson and Holbrook, 2003). This may in part explain why it has such a relatively low plate resistance.

One may, however, speculate that equation (13) points in the direction of the existence of a general allometric scaling law for the sieve plate resistance. Such a law is known to exist for the xylem, where structures similar to sieve plates separate adjacent vascular cells, and where, e.g., Sperry et al. (2005) found proportionality between lumen and end-wall resistance, very similar to our result in equation (13). Further investigations are needed to confirm this hypothesis.

## Discussion and Conclusion

In this paper, we have studied the effect of sieve plates on the hydraulic resistance of phloem sieve tubes. We have derived an analytical expression [equations (10) and (11)] for the resistance based on fact that the flow occurs at low Reynolds numbers and that the pore radii are normally distributed.

Using published data on the structure of sieve plates, we have found an approximately linear relationship between the plate *R_P_* and lumen *R_L_* resistance: *R_P_* ∼ *R_L_*. This implies that the presence of sieve plates increases the hydraulic resistance of the entire sieve tube element by a factor of ∼2.

It should, however, be noted that the current calculations are based on the assumption that sieve tubes are perfectly cylindrical. In reality sieve tubes are often bone shaped with significantly larger sieve plate diameters than lumen diameters, effectively decreasing the sieve plate resistance. Figure 4 nicely illustrates that sieve plate induced flow disturbance occurs in direct vicinity of the plate only. Tube diameter increase close to the sieve plate (see Figure 2 in Knoblauch and Peters, 2010) appears to account for this phenomenon and effectively optimizes tube anatomy for low resistance.

Numerical simulations performed in this study indicate that equation (11) more accurately reflects plate resistance than the equation used in Mullendore et al. (2010). The consequence is that the pressure required to drive flow through the sieve tube system has been underestimated in Mullendore et al. (2010) by about 50%. This increases an often discussed problem in our understanding of phloem function. In some cases, especially in big trees, the turgor pressure generated in sieve tubes appears not to be sufficient to drive flow at standard velocities. Direct measurements of sieve tube pressure using pressure probes glued to aphid stylets, indicated values of 0.5–1.2 MPa in *Salix* (Wright and Fisher, 1980, 1983) and 0.7–1 MPa in *Sonchus* (Gould et al., 2004). Not all of this pressure would be available for flow, since sink tissues also possess turgor. Sink turgor in barley is for example 0.7 MPa (Pritchard, 1996). If sink turgor is similar in *Salix*, little pressure would be available for flow, thus resulting in the need for sieve tubes with extremely high conductivity due to the long stem axis. In addition, it has recently been shown that sieve tube organelles may occupy up to 30% of the sieve element lumen (Froelich et al., 2011) adding further resistance in the sieve tube path. Unfortunately, there are currently no combined data on sieve tube anatomy, flow velocity, and sieve element pressure available. This study provides the theoretical basis for accurate calculations of sieve plate resistance. It will now be important to gather the required data to understand fundamental principles of phloem function.

## Conflict of Interest Statement

The authors declare that the research was conducted in the absence of any commercial or financial relationships that could be construed as a potential conflict of interest.

## Supplementary Materials

The Supplementary Material for this article can be found online at http://www.frontiersin.org/Plant_Biophysics_and_Modeling/ 10.3389/fpls.2012.00151/abstract

## Figures and Tables

**Figure A1:**
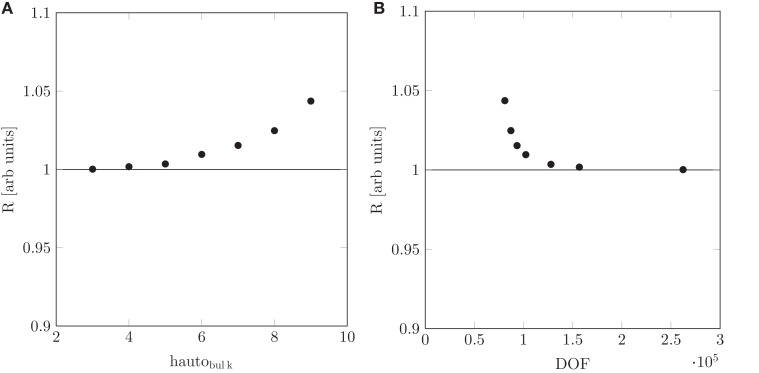
**Mesh convergence plots**. Hydraulic resistance *R* plotted as a function of **(A)** hauto_bulk_ and **(B)** the number of degrees of freedom DOF with hauto_pore_ = 4 and *N*_sweep_ = 10.

## Appendix

### A Numerical methods

Following the procedure outlined in Figure 3, we set up the numerical simulation in the commercial finite-element method software package comsol Multiphysics 3.5a. For symmetry reasons, it suffices to solve the problem in half the sieve element as sketched in Figure 1A. The velocity and pressure fields was subsequently determined by numerical solution of the Stokes and continuity equations

$$\begin{array}{rc} \nabla p=\nabla^{2}u, & \text{(A1)}\\ \nabla \cdot u=0 & \text{(A2)} \end{array}$$

where ***u***(*x*, *y*, *z*) = (*u*, *v*, *w*). To simplify the mathematical expressions we are using non-dimensional variables. The explicit scalings are: Lengths are given by the sieve element radius *r*, velocities are given by the characteristic flow velocity *u*_0_. Moreover, pressure is given in terms of the shear-stress pressure *p*_0_ = η*u*_0_/*r*. The velocity boundary condition at the walls Ω*_w_*, is that the velocity ***u*** is zero

(A3) $$\text{u}\left(x,y,z\right)=0,\;\text{for}\left(x,y,z\right)\in \Omega_{w}.$$

Assuming that the sieve element is oriented along the *x*-axis, the velocity boundary condition at the inlet Ω*_i_* is,

(A4) $$\text{u}=\left({\left(y-1\right)}^{2}+{\left(z-1\right)}^{2},0,0\right)\;\text{for}\left(x,y,z\right)\in \Omega_{i}.$$

The outlet boundary conditions, corresponding to boundary conditions at the center plane of the sieve plate Ω*_o_*, are

(A5) $$v=w=\frac{\partial u}{\partial x}=p=0\;\text{for}\left(x,y,z\right)\in \Omega_{o}.$$

Numerically, the main challenge faced when solving equations (14–18) close to the sieve plates is to keep the number of degrees of freedom (DOF), proportional to the number of mesh elements and thus required computational time, low while accurately resolving the flow details. To achieve this, the mesh was generated using a linearly swept mesh with *N*_sweep_ nodes along the *x*-axis inside the sieve pores. This mesh was then connected to a coarser mesh far away from the plate. In this procedure the comsol hauto mesh generation procedure was used throughout, with characteristic mesh sizes hauto_pore_ and hauto_bulk_ corresponding to the pore and bulk sieve tube regions respectively. Note that smaller values of hauto generate finer meshes with more elements (COMSOL AB, 2008) To test solution convergence, we simulated the flow through 4 × 4 pores in a periodic rectangular domain. We varied *N*_sweep_ from 10 to 30 and hauto_pore_ and hauto_bulk_ from 3 to 8. As shown in Figure A1, numerically computed resistances converged rapid when *N*_sweep_ = 10 and hauto_pore_ = 4, and a reasonably low number of DOF was obtained with hauto_bulk_ = 5, the value used for the simulations presented in the present paper. From Figure A1, we observe that a conservative estimate of the relative error in the numerically determined resistance is 5%.

### B Caption for Supplementary Movie I

**Table A1:** **Sieve tube element data from Mullendore et al., 2010, (1–5) to Thompson and Holbrook, 2003, (6–19)**.

No.	Species	*r*[μm]	ℓ[μm]	${\bar{r}}_{p}\pm \sigma_{p}[\mu \text{m}]$	ℓ_*p*_[μm]	*N*
1	*Cucurbita maxima*	25.65 ± 2.97	341 ± 77	2.54 ± 0.86	1.27 ± 0.29	54.8 ± 11.9
2	*Phaseolus vulgaris*	10.13 ± 1.13	140 ± 38	0.73 ± 0.24	0.43 ± 0.11	95.4 ± 31.7
3	*Solanum lycopersicum*	10.70 ± 1.40	130 ± 90	0.61 ± 0.15	0.52 ± 0.12	121.3 ± 30.0
4	*Ricinus communis*	16.22 ± 1.60	255 ± 122	0.52 ± 0.14	0.24 ± 0.05	371.9 ± 79.0
5	*Phyllostachys nuda*	11.60 ± 1.00	1052 ± 244	0.61 ± 0.13	0.39 ± 0.10	105.6 ± 12.7
6	*Pinus strobus*	10.9	1580	0.35	2.5	720
7	*Festuca arundinacea*	3	100	0.3	0.5	33
8	*Beta vulgaris*	5	200	0.1	0.4	1250
9	*Glycine max* (petiole)	4.2	125	0.35	1.1	58
10	*Glycine max* (stem)	6.6	156	0.6	1.2	81
11	*Glycine max* (root)	5.1	137	0.5	1.0	60
12	*Gossypium barbadense*	11	210	0.5	1.0	160
13	*Sabal palmetto*	18	700	0.95	0.5	287
14	*Yucca flaccida*	10	460	0.26	0.4	1746
15	*Robinia pseudoacacia*	10	180	1.25	0.5	21
16	*Tilia americana*	15	350	0.6	0.8	625
17	*Ulmus americana*	18	190	2.0	1.0	50
18	*Cucurbita melopepo*	40	250	2.4	0.5	120
19	*Vitis vinifera*	18	500	0.7	3.5	661

*Sieve tube radius *r*, sieve tube element length ℓ, sieve pore average radius ${\bar{r}}_{p}$ sieve pore radius standard deviation σ*_p_*, sieve plate thickness *ℓ_p_*, and number of pores per plate *N**.
